# 07. Recombinant Zoster Vaccine (RZV) Second-Dose Completion in Adults Age 50‒64 Years in the United States

**DOI:** 10.1093/ofid/ofab466.210

**Published:** 2021-12-04

**Authors:** Jessica Leung, Elizabeth B Gray, Tara Anderson, Sarah M Sharkey, Kathleen L Dooling

**Affiliations:** 1 CDC, Atlanta, GA; 2 Division of Health Informatics and Surveillance, Center for Surveillance, Epidemiology, and Laboratory Services, Centers for Disease Control and Prevention, Atlanta, Georgia; 3 Division of Viral Diseases, National Center for Immunization and Respiratory Diseases, Centers for Disease Control and Prevention, Atlanta, Georgia; 4 IQVIA Government Solutions, Information and Analytics, Atlanta, Georgia

## Abstract

**Background:**

In 2018, CDC recommended a highly efficacious adjuvanted recombinant zoster vaccine (RZV, Shingrix) as a 2-dose series for prevention of herpes zoster (HZ) for immunocompetent persons age ≥50 years, with the 2^nd^ dose recommended 2–6 months after the 1^st^ dose. Among Medicare beneficiaries, 2-dose series completion 6 months and 12 months post initiation was 78% and 86%, respectively. Here we estimate the proportion of adults age 50–64 years who completed the 2-dose RZV series within 6 or 12 months after receiving their 1^st^ dose, by using two administrative claims databases.

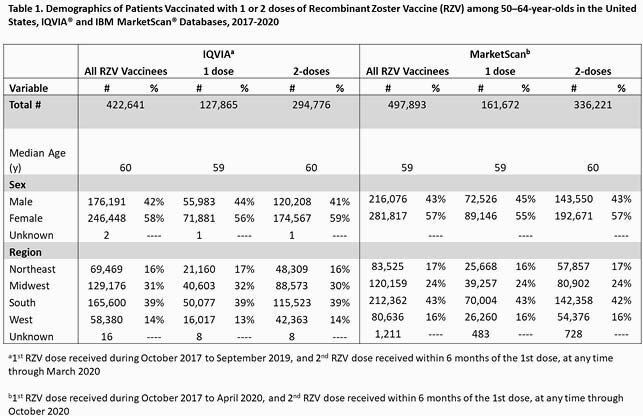

**Methods:**

We used medical and pharmaceutical claims data from October 2017‒March 2020 IQVIA® PharMetrics Plus and October 2017‒October 2020 IBM® MarketScan® databases. RZV vaccination was defined using Current Procedural Terminology and National Drug Codes. We allowed for sufficient follow-up time by examining 1^st^ doses given at least 6 or 12 months prior to the end of the study period in both databases. Place of administration was available in IQVIA data.

**Results:**

Among persons age 50‒64 years, in IQVIA and MarketScan, 70% and 68% received their 2^nd^ RZV dose within 6 months, respectively, and 79% and 81% received their 2^nd^ dose within 12 months, respectively. The median age of 1^st^ dose of RZV vaccination was 60 years and ~60% were female [**Table 1**]. When the 2^nd^ dose was administered within 12 months, the median interval between 1^st^ and 2^nd^ doses was 104 and 98 days in the IQVIA and MarketScan databases, respectively. Characteristics by age, sex, or region were similar in persons who received 1 RZV dose vs. 2 RZV doses [**Table 1**]. Among those who received only 1 RZV dose with at least 12 months of follow-up time, 55% of vaccinations occurred at ambulatory medical provider offices and 40% at pharmacies; among 2 doses recipients, 33% of vaccinations occurred at provider offices and 62% at pharmacies.

**Conclusion:**

Among 50‒64-year-olds, 2-dose RZV series completion was ~70% within 6 months and 80% within 12 months of initiation. The findings were similar across two administrative claims databases. Availability of RZV at pharmacies has potentially helped to increase RZV 2^nd^ dose completion rates.

**Disclosures:**

**All Authors**: No reported disclosures

